# Development of a Novel *Pseudoperonospora cubensis*-Specific LAMP Assay for Rapid Field Detection and Surveillance of Cucurbit Downy Mildew

**DOI:** 10.3390/jof12080626

**Published:** 2026-08-20

**Authors:** Sumyya Waliullah, Md Tahsinul Anwar, Emran Ali

**Affiliations:** 1 Department of Biological Sciences, Alcorn State University, Lorman, MS 39096, USA; 2 Department of Agriculture, Alcorn State University, Lorman, MS 39096, USA

**Keywords:** *Pseudoperonospora cubensis*, cucurbit downy mildew, loop-mediated isothermal amplification, molecular diagnostics, early detection, spore trap, field-deployable

## Abstract

Cucurbit downy mildew, caused by the oomycete *Pseudoperonospora cubensis*, is a devastating disease of cucurbit crops requiring rapid detection for effective management. Current molecular diagnostics often rely on complex laboratory infrastructure, limiting their on-site applicability. To address this limitation, we developed a highly specific, field-deployable loop-mediated isothermal amplification (LAMP) assay targeting the *P. cubensis*-specific c2555.2e1 marker gene. The assay’s optimal reaction conditions were determined, and its specificity was evaluated using closely related oomycetes, including *P. humuli* and *P. belbahrii*, alongside other fungal species. Assay sensitivity was compared to conventional PCR using a pathogen DNA dilution series, and diagnostic efficacy was validated using field-collected winter squash samples suspected of infection and environmental spore-trap samples. The optimal LAMP reaction conditions were 71 °C for 60 min. The assay accurately amplified *P. cubensis* with no observed cross-reactivity. Sensitivity analysis demonstrated a detection limit of 100 pg/µL, and was at least 10-fold more sensitive than conventional PCR. Furthermore, the LAMP assay exhibited greater detection sensitivity than conventional PCR when analyzing environmental and field samples. This rapid and sensitive LAMP assay provides a rapid and highly sensitive tool for on-site diagnostics, offering a crucial advancement for the early detection and management of cucurbit downy mildew.

## 1. Introduction

Downy mildews are plant diseases caused by obligate oomycete pathogens belonging to the family *Peronosporaceae*. These pathogens infect various vegetable crops, resulting in devastating crop loss each year. Cucurbit downy mildew (CDM) is one of the most economically damaging types of downy mildew which is caused by *Pseudoperonospora cubensis*. This notorious pathogen is known to impact over 60 species belonging to 20 genera in the Cucurbitaceae family worldwide [1,2,3].

The distribution of *P. cubensis* relies on the presence of susceptible host species [4]. This pathogen overwinters in southern regions of the United States characterized by warm and humid conditions, moving north with the summer months [5]. Disease outbreak occurs with aerially dispersed sporangia from subtropical overwintering sources or infected greenhouse-grown crops [5,6]. Previously, *P. cubensis* sporangial loads were surveilled morphologically using Burkard spore traps and microscopy [7]. However, morphological similarities between *P. cubensis* and *Pseudoperonospora humuli*, the causal agent of hop downy mildew (HDM), might lead to inaccurate estimations of the pathogen’s identity based on morphology alone [1,8].

Management of CDM was previously effective with resistant cultivars, a result of decades-long breeding programs, leading back to the 1940s to 1960s [9,10,11,12]. These practices were no longer enough after a large outbreak in 2004 and 2005 that was attributed to a new, more virulent strain of *P. cubensis* [13]. Years with favorable environmental conditions and no fungicide use have resulted in up to 100% crop loss [9,12,13]. The economic impact of *P. cubensis* has been significant; 2004 brought a loss of $16 million to the eastern US [14]. Given the importance of managing this disease, a forecasting system, CDM ipmPIPE, in collaboration with the iPiPE Cooperative Agricultural Project, has been generated in an effort to track the movement of CDM and is estimated to have saved over $6 million in fungicide use for the states of Georgia, North Carolina and Michigan, which account for one quarter of all cucumber production in the United States [15]. Nonetheless, alerts based on reported outbreaks of commercial cucurbits do not account for other sources, including greenhouse-grown infection, wild cucurbits reservoir or seedborne infection [16,17,18,19]. In addition, the infectious nature and increased fungicide resistance of this pathogen require an improved forecasting alert system through early detection strategies [20].

Therefore, researchers have developed several advanced diagnostic assays to detect *P. cubensis* in earlier stages of infection alongside the morphological identification [21,22,23]. Several PCR-based assays were developed recently to detect downy mildew pathogens such as *P. cubensis*, *P. humuli*, or *P. belbahrii* based on conserved regions, namely, internal transcribed spacer (ITS) or mitochondrial genes [21,23,24,25]. However, targeting conserved regions might result in nonspecific amplification of closely related species, which is not suitable for monitoring airborne inoculum [24]. Hence, several species-specific quantitative PCR assays were further designed to improve the detection and monitoring of airborne *P. cubensis* sporangial concentrations [26,27]. Recently, Withers et al. [22] identified seven different *P. cubensis*-specific PCR markers, utilizing a comparative genomics strategy to differentiate it from the closely related species *P. humuli*. While these markers are highly specific, traditional PCR is not field-deployable. Conversely, recent efforts have successfully developed field-deployable LAMP assays for *P. cubensis* targeting genes such as cellulose synthase 2 (CesA2) [28]. However, these isothermal assays were not validated against *P. humuli*, leaving a critical gap in ensuring diagnostic accuracy for environmental monitoring where these sister species cannot be distinguished morphologically. Therefore, to achieve both rapid field-deployability and absolute specificity, we have developed a *P. cubensis*-specific isothermal quick detection assay leveraging the c2555.2e1 gene reported by Withers et al. [22].

The loop-mediated isothermal amplification (LAMP) approach offers a robust alternative to conventional microscopy and PCR-based diagnostics by mitigating their inherent logistical limitations [29]. LAMP is exceptionally advantageous for CDM management because its simplicity, affordability, and portability facilitate the identification of the pathogen during the onset of infection [29,30,31]. By utilizing a set of four to six target-specific primers, LAMP achieves exceptional specificity and has been shown to be 10 to 100 folds more sensitive than standard PCR. It can successfully detect trace amounts of nucleic acids (<1 pg/µL) irrespective of the original sample matrix [30,31]. Consequently, our objective in this study was to design a highly specific LAMP assay capable of detecting *P. cubensis* in established infections, asymptomatic field plants, and airborne inoculum captured via spore traps. The ability to test many samples with a cost-effective, highly-sensitive and rapid assay would provide greater accuracy for tracking CDM and, therefore, better management of this devastating disease.

## 2. Materials and Methods

### 2.1. Fungal and Plant Materials Used in This Study

Isolates of known oomycete pathogens including *P. cubensis* and other closely related species used in this study for genomic DNA extraction are listed in Table 1. *P. cubensis*, *P. humuli* and *Peronospora belbahrii* genomic DNA were sourced from the Vegetable Laboratory, Department of Entomology and Plant Pathology, North Carolina State University. Additional oomycetes DNA samples were collected from the Soybean Pathology Laboratory, Ohio State University. One known ascomycete, *Blumeria graminis*, was isolated from a rescue grass sample for genomic DNA extraction at Plant Molecular Diagnostic Lab, University of Georgia (UGA-MDL). *P. cubensis*-infected field-grown cucurbits were sampled from two separate field sites in Tift County, Tifton, GA and Lowndes County, Valdosta, GA. Burkard spore-trapped *P. cubensis* sporangial DNA samples were received from the Vegetable Laboratory, Department of Plant, Soil and Microbial Sciences, Michigan State University. Healthy control samples were obtained from greenhouse-grown squash in UGA-MDL for genomic DNA extraction.

### 2.2. DNA Extraction

For DNA extraction of *P. cubensis* sporangia from field-grown samples and *Blumeria graminis*-infected rescue grass sample, DNeasy^®^ Plant Mini Kit (QIAGEN, Germantown, MD, USA) was utilized with a slight modification to the kit protocol. Briefly, sporangia were gently scraped from infected cucurbits (cucumber and squash) into 15 mL centrifuge tubes and resuspended in 500 μL of Buffer AP1 and 5 μL of RNase A. The sporangial suspension was then transferred into MP Biomedicals Lysing Matrix A impact-resistant 2 mL tubes for homogenization. The samples were homogenized using FastPrep™ FP120 (Thermo Electron Corporation, Waltham, MA, USA) at a speed setting of 6.0 for 40 s and repeated twice if larger tissue pieces were still visible. The remainder of the protocol was followed using a DNeasy^®^ Plant Mini Kit with a minor modification to the kit protocol. For DNA extraction from healthy control samples, the above-mentioned protocol was followed, except healthy leaf tissues were homogenized using liquid nitrogen. One hundred milligrams of the homogenized leaf tissue with buffer and RNase A were then transferred to the Lysing Matrix A tubes and vortexed at setting 7–10 for 5–10 s. The remainder of the protocol was followed using DNeasy^®^ Plant Mini Kit with modification. Total DNA yield and purity were determined with a NanoDrop spectrophotometer (NANODROP LITE, Thermo Scientific, Waltham, MA, USA) and either used immediately or stored at −20 °C.

### 2.3. Sample PCR for Sequencing and Candidate Gene Selection

*P. cubensis* genomic DNA (Table 1) obtained from the Vegetable Laboratory, Department of Entomology and Plant Pathology, North Carolina State University, was amplified and sequenced with candidate marker genes for LAMP primer design. PCR amplification was performed using the published PCR primers (Table 2) of two highly specific diagnostic markers for *P. cubensis*, c2555.2e1 and c2555.3e7, for candidate gene selection [21]. Primers were synthesized by Sigma-Aldrich (Sigma-Aldrich, St. Louis, MO, USA), dissolved in PCR-grade water (Sigma-Aldrich) to produce 100 µM solutions, and stored at −20 °C. PCR reactions were performed on a thermocycler (Biorad-96 well T100™, Biorad, Hercules, CA, USA) using EconoTaq PLUS GREEN 2X Master Mix (Lucigen, Madison, WI, USA). The PCR reaction contained 2 μL of sample DNA at a concentration of 10 ng/μL with 0.3 µM of both the forward and reverse primers, 15 μL of 2X Econotaq master mix (Lucigen, Madison, WI, USA), and deionized PCR grade water to a final volume of 30 μL. PCR conditions were as follows: an initial denaturation step at 94 °C for 3 min, followed by 35 cycles of 30 s at 94 °C, 30 s at 55 °C, 30 s at 72 °C and a final extension at 72 °C for 5 min [21]. The amplified PCR product was purified with Quantum Prep PCR Kleen Spin Columns (Bio-Rad Laboratories, Hercules, CA, USA) with slight modifications to the kit protocol and submitted for sequencing (Retrogen, San Diego, CA, USA). The draft sequences from forward and reverse primers were then imported into Geneious Prime 2019.2.3 (Biomatters Ltd., Auckland, New Zealand) software and cleaned based on the chromatogram files.

### 2.4. LAMP Primer Design

We utilized Primer Explorer V5 (http://primerexplorer.jp/lampv5e/; accessed on 1 August 2026) to construct LAMP primer candidates from the obtained *P. cubensis* c2555.2e1 and c2555.3e7 sequences. To ensure target specificity, candidate sequences were analyzed using the NCBI Basic Local Alignment Search Tool (BLAST; BLAST+ version 2.12.0) against the nucleotide (nr/nt) and relevant whole-genome shotgun (WGS) databases using standard parameters. Three distinct primer sets were designed from these genes, each comprising two outer primers (F3 and B3), two inner primers (FIP and BIP), and two loop primers (LF and LB) (Supplementary File S1). Oligonucleotides were synthesized by Sigma-Aldrich (St. Louis, MO, USA), reconstituted to 100 µM in qPCR-grade water, and maintained at −20 °C. Following initial laboratory screening against *P. cubensis* (C1) and *P. humuli* (H1) genomic DNA, the most robust primer set was selected for all subsequent evaluations (Figure S1). All LAMP reactions were performed independently three times, and consistent results were obtained in all experiments.

### 2.5. Reaction Conditions for LAMP

We performed the LAMP assay utilizing the LavaLAMP™ DNA Master Mix (Lucigen, Middleton, WI, USA). Each 25 µL reaction volume consisted of 12.5 µL of the 2× master mix, 1.0 µL of Lucigen Green Fluorescent Dye, 1.0 µL of template DNA, and nuclease-free water. The primer concentrations were adjusted to 1.6 µM for both FIP and BIP, 0.8 µM for the loop primers (LF and LB), and 0.2 µM for the outer primers (F3 and B3) (Table 2). Assays were run on the Genie^®^ III platform (OptiGene, Horsham, UK), and results were evaluated via the companion Genie Explorer software. The thermal profile included an initial 90 °C heating step for 3 min, followed by isothermal amplification at 71 °C for 60 min. The run concluded with an annealing curve generated by cooling from 98 °C to 80 °C at a ramp rate of 0.05 °C per second.

### 2.6. Optimization of LAMP Conditions 

The LAMP assay was performed using previously optimized primer concentration and duration (M&M Section 2.5) [31,32]. To determine the optimal reaction temperature for the detection of *P. cubensis*, isolate C1 genomic DNA was used as a template. A gradient LAMP was performed with a temperature range of 66 to 73 °C using 1.0 ng/µL *P. cubensis* C1 genomic DNA diluted from the initially extracted DNA sample. Uninfected healthy squash leaf samples and the reaction mix with molecular grade water were used as a healthy and negative control. The reaction was performed using LavaLAMP™ DNA Master Mix (Lucigen, Middleton, WI, USA) according to the reaction mixture and protocol described in the methods section. For real-time amplification, the Genie^®^ III (OptiGene, Horsham, WS, UK) instrument was utilized. For optimizing reaction temperature, two parameters obtained from the Genie^®^ III were evaluated: amplification time (Tiamp) and amplicon annealing temperature (Ta), which were recorded in a table (Table S1). The temperature with the shortest amplification time (Tiamp) and peak value of the melting curves (Ta) was considered the optimum temperature for the assay (Table S1).

### 2.7. Specificity Analysis of LAMP

**Figure 1:**
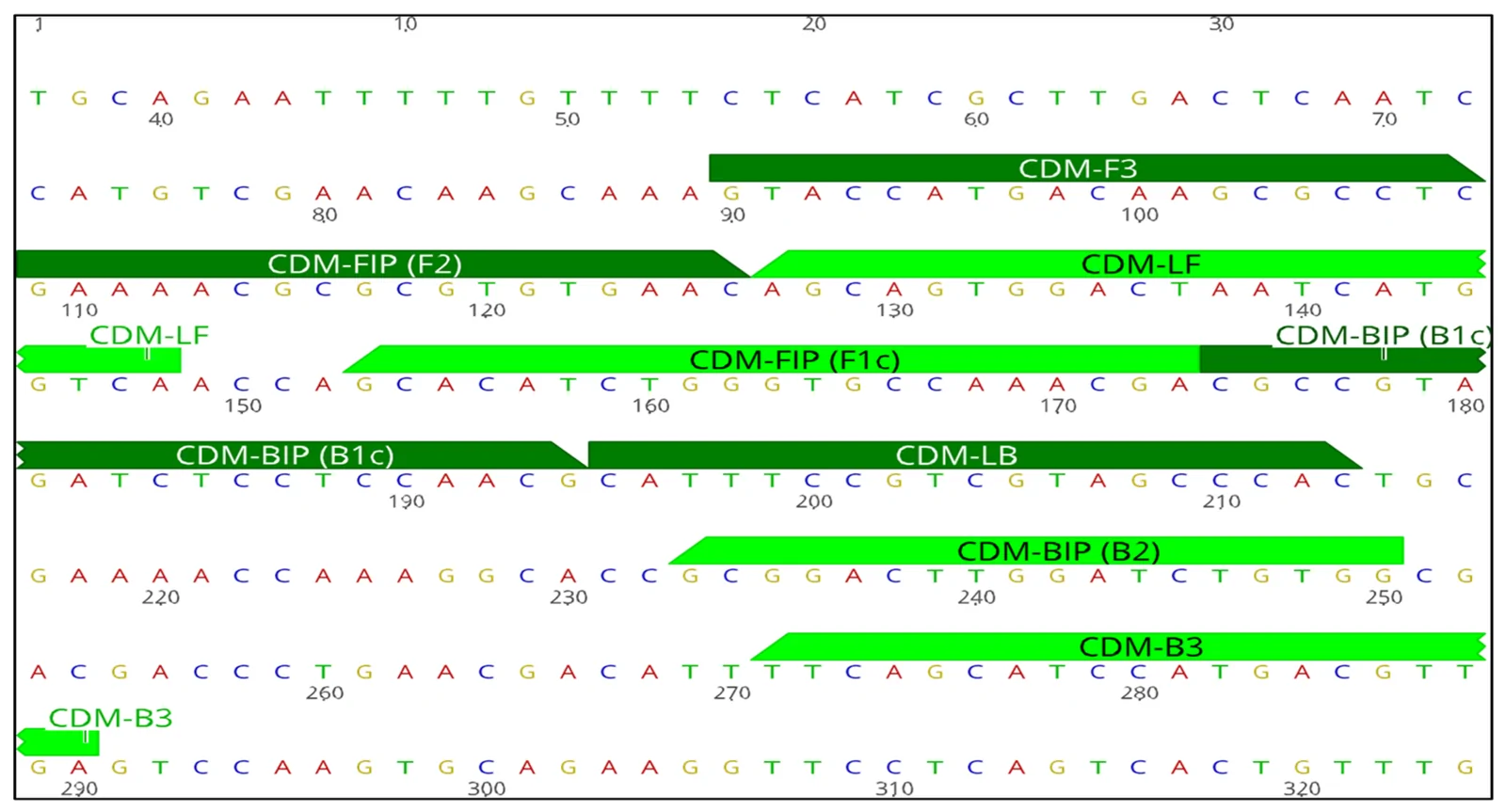
Location of LAMP primer sets (F3, B3, FIP [F1c-F2], and BIP [B1c-B2]) targeting partial sequence of *P. cubensis* c2555.2e1 gene sequence. FIP is a hybrid primer comprising the F1c and F2 sequences, whereas BIP is a hybrid primer comprising the B1c and B2 sequences. Arrows indicate the extension direction.

**Figure 2:**
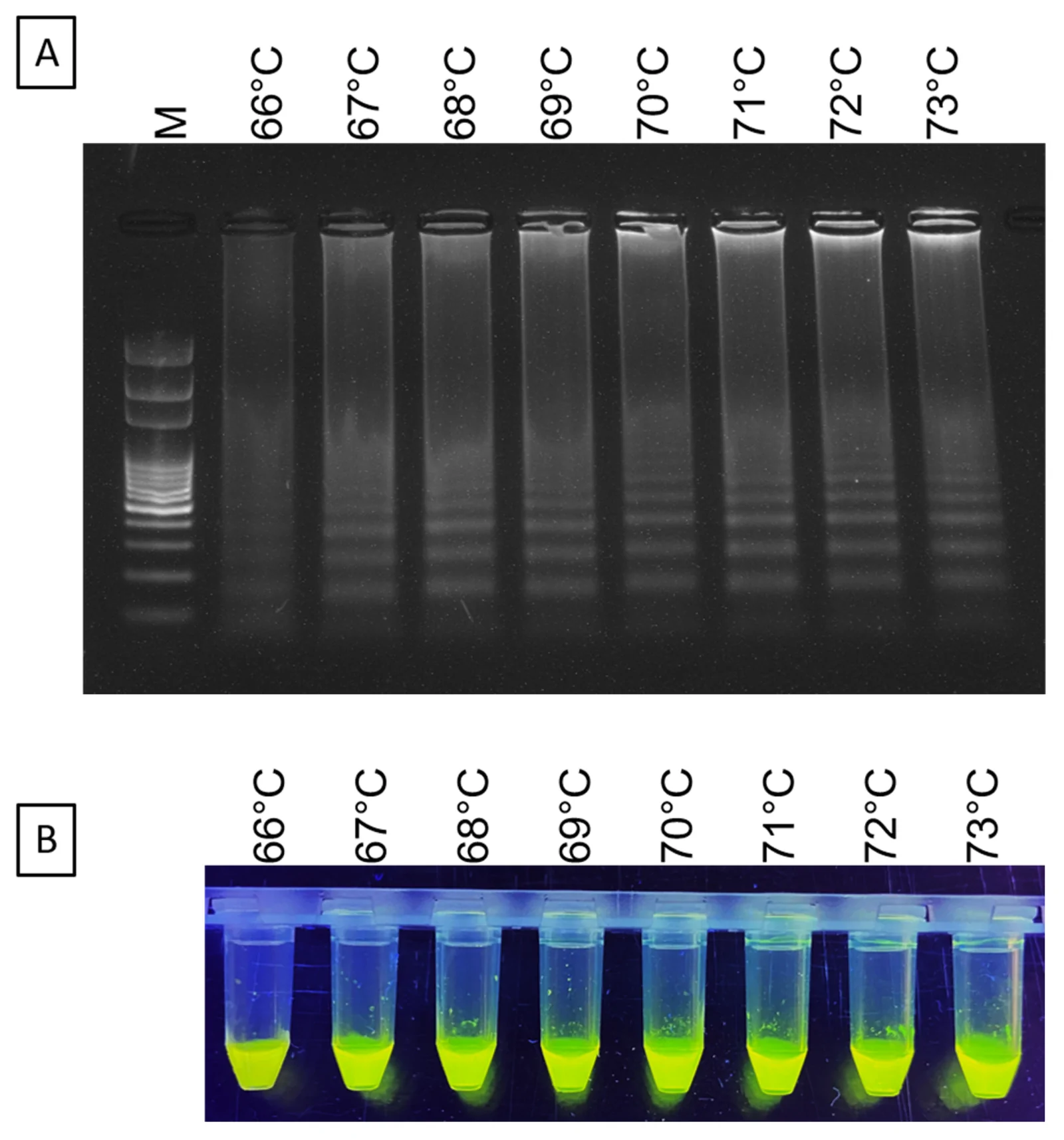

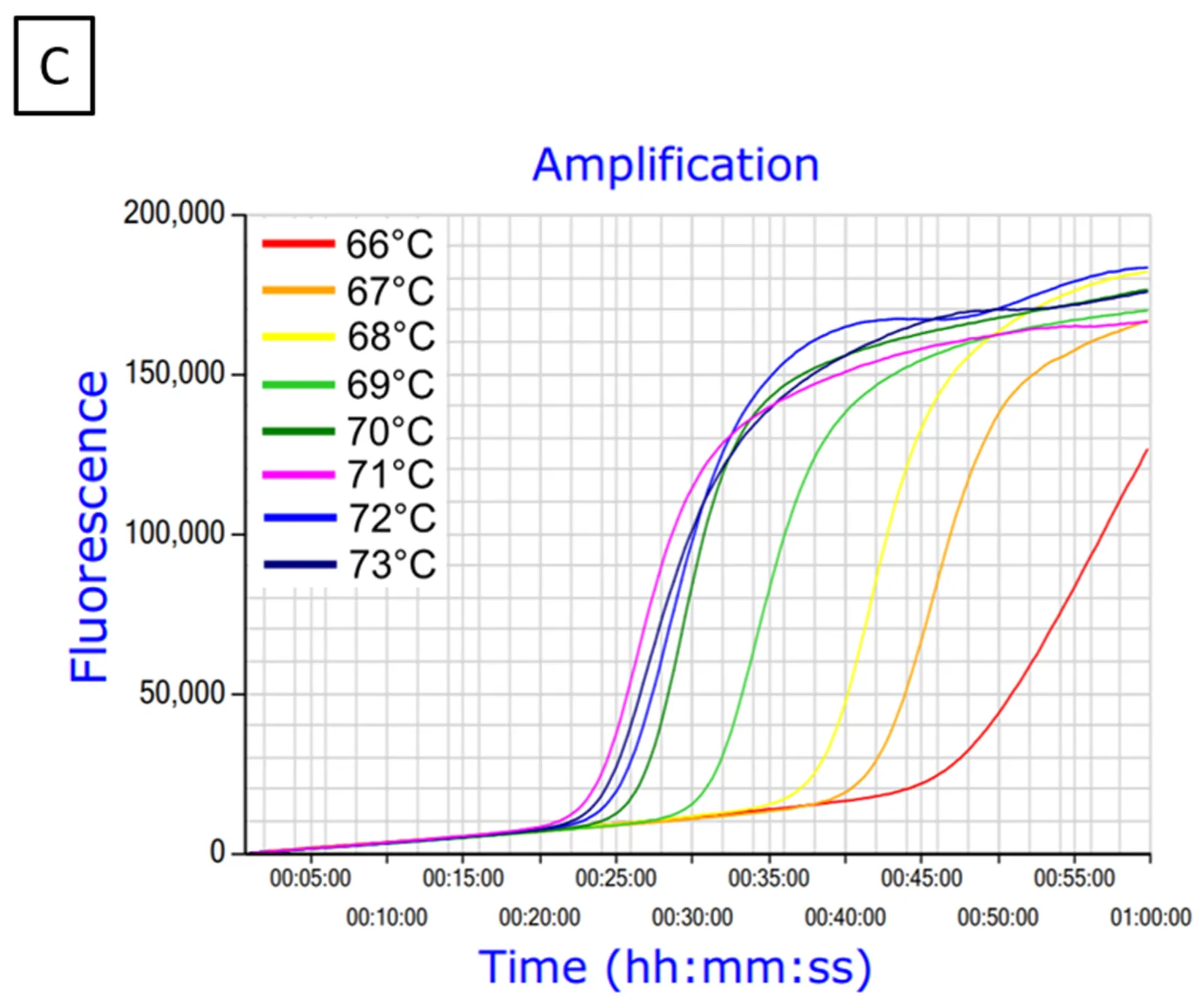
Optimization of the LAMP reaction temperature for *Pseudoperonospora cubensis*. The LAMP assay was performed using a temperature gradient from 66 to 73 °C. Amplification products were analyzed by (**A**) 1.0% agarose gel electrophoresis, (**B**) SYBR™ Green I staining under UV light, and (**C**) real-time amplification using the Genie^®^ III system, M: 100-bp DNA ladder. The corresponding annealing temperatures (Ta) of the LAMP amplicons were 82.3 °C for reactions at 66 °C, 82.4 °C for 67–69 °C, 82.5 °C for 70 °C and 73 °C, and 82.1 °C for 71–72 °C, confirming the amplification of the same specific target across all reaction temperatures tested.

Genomic DNA extracted from isolates of two oomycete pathogens closely related to *P. cubensis*, *P. humuli* and *Peronospora belbahrii*, was utilized to verify the specificity of the LAMP assay (Figure 1). An additional specificity test was carried out with isolates from five other known oomycete pathogens, *Phytophthora sojae*, *Phytopythium vexans*, *Pythium irregulare*, *Pythium dissotocum* and *Phytophthora sansomeana*, and one ascomycete pathogen, *Blumeria graminis* (Figure 2). LAMP assays with diluted 1 ng/μL genomic DNA were performed according to the methods section. DNA from healthy squash leaves was used as a healthy control, and nuclease-free H_2_O was used as the negative control. PCR reactions with universal ITS1/ITS4 primers were performed as a positive control to confirm the successful extraction of amplifiable DNA and to verify the absence of amplification inhibitors in the samples.

### 2.8. Comparative Sensitivity Analysis of LAMP

The sensitivity of the LAMP assay for detecting *P. cubensis* DNA was evaluated using DNA from isolate C1 at concentrations ranging from 10 ng/µL to 0.001 fg/µL. To compare the sensitivity of the LAMP assay with conventional PCR, two PCR primer sets previously used for sequencing the marker genes (Table 2) were evaluated. The more efficient PCR primer set c2555.3e7_F/c2555.3e7_R used to detect *P. cubensis* infection was further exploited for comparative sensitivity analysis and field-collected-sample infection. The assay was executed according to the method described above. Nuclease-free H_2_O was used as the negative control. The sensitivity assay was performed independently three times using the same serial DNA dilutions, and consistent results were obtained across all experiments.

### 2.9. Evaluation of Infected Field Samples Using LAMP Assay

Winter squash leaf samples suspected of *P. cubensis* infection were collected from several fields in Tift and Lowndes counties, Georgia, USA, for LAMP analysis. Twenty winter squash leaf samples from suspected symptomatic plants were obtained from the two counties and were tested using PCR and LAMP methods. Total DNA was extracted from collected leaf material using the protocol described above (see Section 2.2). Healthy squash leaf tissues were used as the healthy control and deionized water as a negative control. All amplification reactions were carried out using 10 ng of total DNA. LAMP amplification was performed using Genie^®^ III (OptiGene, Horsham, WS, UK) for real-time observation. The Genie^®^ III amplified products were separated and analyzed on a 1.0% agarose gel and visualized by adding SYBR™ Green 1 nucleic acid gel stain for further confirmation. Samples were considered positive only when they exhibited a characteristic sigmoidal amplification curve with the expected annealing temperature (Ta ≈ 82.1 °C), supported by agarose gel electrophoresis and/or SYBR™ Green I visualization. Samples showing weak, delayed, or inconsistent amplification that did not meet all of these criteria were classified as suspected positive, whereas samples showing no amplification were classified as negative. The positive and negative results obtained from each method with respective annealing temperature were compared and recorded in a table (Table 3). Results were also visualized for comparative sensitivity analysis between LAMP and PCR assays to detect *P. cubensis* infection from suspected field-grown samples.

## 3. Results

### 3.1. Candidate Marker Gene Selection and LAMP Primer Designing

Among the seven highly specific diagnostic molecular marker genes (c10851.1e0, c2555.2e1, c2555.3e7, c3155.4e9, c52.12e13, c572.6e19, and c2183.6e2) reported by Withers et al. [22], which could be used to distinguish *P. cubensis* from its sister species *P. humuli*, two single-copy genes, c2555.2e1 and c2555.3e7, showing no cross-reactivity [21] were selected as our LAMP candidate marker genes. Sanger sequences of the amplified PCR products with the published PCR primers (Table 2) were obtained for LAMP primer designing. Following a BLAST search of the two sequences against the whole NCBI nucleotide collection (nr/nt) database and closely related oomycetes and ascomycetes whole genome shotgun contigs (wgs), the only match found was with *P. cubensis* isolate MSU-1 wgs (GenBank accession number: AHJF01002473.1). The isolate contig psc_contig_2555 sequence matched with c2555_2e1 marker with 99.86% and c2555_3e7 marker with 100%. Three sets of LAMP primers were designed out of the two sequences using Primer Explorer version 5 (http://primerexplorer.jp/lampv5e/; accessed on 1 August 2026) (Supplementary File S1) for laboratory validation. LAMP amplification using 1.0 ng/µL genomic DNA from *P. cubensis* C1 and the closely related species *P. humuli* H1 revealed that primer set 2 was the most efficient of the three sets, specifically amplifying *P. cubensis* DNA with the highest fluorescence signal and amplification rate and producing a single distinct peak ratio and amplification rate and with a single peak point (Figure S1). Therefore, primer set 2 was selected for all subsequent LAMP experiments. Although the initial screening was performed using representative isolates, future evaluation using a larger collection of *P. cubensis* and *P. humuli* isolates would provide a more comprehensive assessment of primer performance and specificity.

### 3.2. Optimization of LAMP Conditions for P. cubensis Detection

The optimal temperature for the LAMP assay was determined using genomic DNA from *P. cubensis* isolate C1 at a concentration of 1.0 ng/µL. Targeting *P. cubensis* marker gene c2555.2e1, six newly synthesized LAMP primers, including F3, B3, FIP [F1c-F2], BIP [B1c-B2], LF and LB, designed by Primer Explorer version 5 (Figure 1, Table 2) were utilized to optimize the temperature condition. When the LAMP assay was performed over a temperature gradient of 66–73 °C, 71 °C produced the optimal amplification, with the shortest amplification time (Tiamp) of 25 min and a specific amplicon annealing temperature (Ta) of 82.1 °C (Figure 2, Table S2). The optimum 10× formulation of the LAMP primer mix was determined according to the findings from previous studies with a concentration of 0.2 μM for each of primers F3 and B3, 0.8 μM for each of primers LF and LB, and 1.6 μM for each of primers FIP and BIP [30,31]. A reaction time of 60 min was adopted based on the manufacturer’s recommendations and previous validated LAMP protocols [31,32]. Accordingly, the LAMP assay was conducted at 71 °C for 60 min throughout this study.

### 3.3. Specificity of LAMP Detection of P. cubensis

**Figure 3:**
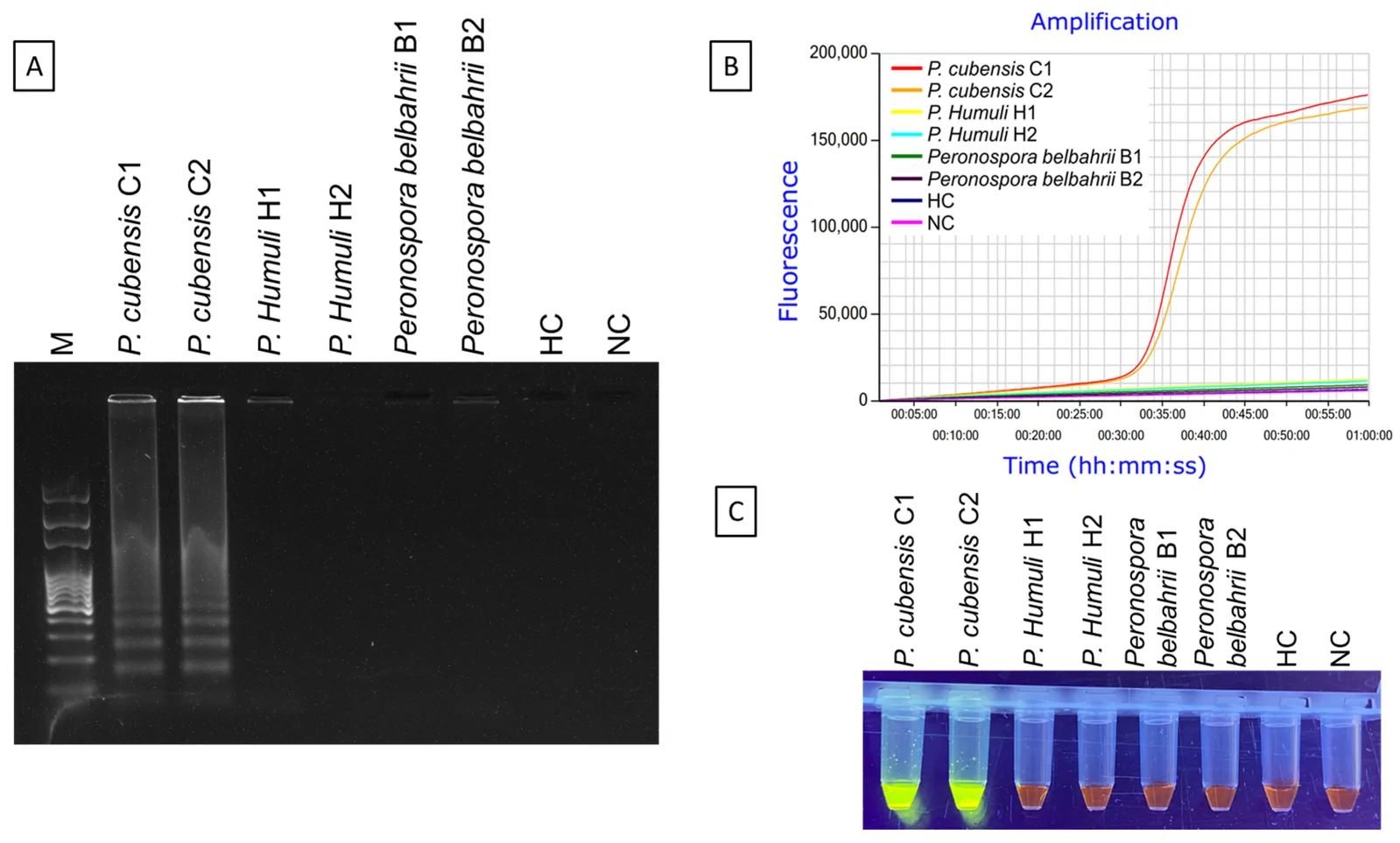
Specificity analysis of the LAMP assay for the detection of *P. cubensis* using DNA of two isolates of *P. cubensis* and two other closely related pathogens, *P. humuli* and *Peronospora belbahrii*. Specific amplification of *P. cubensis* was observed while running the amplified LAMP product using agarose gel electrophoresis (**A**), real-time amplification using Genie^®^ III (**B**) or visual inspection with SYBR™ Green 1 DNA gel staining agent (**C**), whereas the other two pathogens showed no amplification. Here, M: 100 bp DNA ladder; HC: healthy squash leaves as healthy control; NC: nuclease-free H_2_O as a negative control. The fluorescent-green-colored products could be visualized after adding SYBR™ Green 1 DNA gel staining agent to the end product under UV light, while negative reactions remained orange in color. Positive amplification reactions were confirmed by a consistent amplicon annealing temperature (Ta) of 82.1 °C.

**Figure 4:**
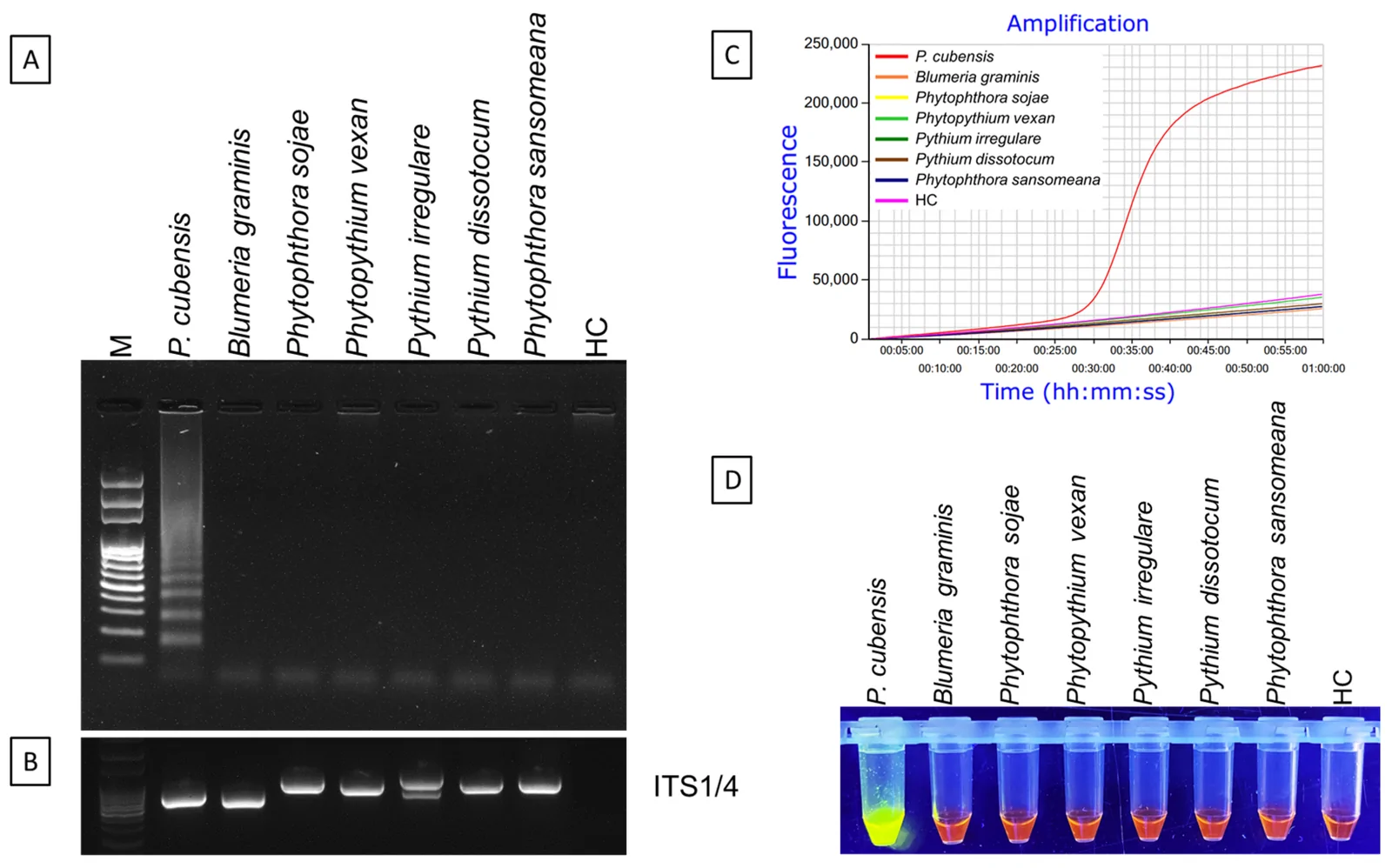
Further specificity analysis of the LAMP assay for the detection of *P. cubensis* using DNA from *P. cubensis*, five other oomycete pathogens (*Phytophthora sojae*, *Phytopythium vexans*, *Pythium irregulare*, *Pythium dissotocum*, and *Phytophthora sansomeana*), and one ascomycete pathogen (*Blumeria graminis*). LAMP products were analyzed by (**A**) agarose gel electrophoresis, (**B**) real-time amplification using the Genie^®^ III system, (**C**) amplification rate analysis using the Genie^®^ III system, and (**D**) visual detection using SYBR™ Green I nucleic acid gel stain under UV light. Specific amplification was observed only for *P. cubensis*, whereas no amplification was detected for the other pathogens or the negative control. M: 100-bp DNA ladder; NC: nuclease-free H_2_O used as the negative control. In panel (**D**), positive *P. cubensis* reactions exhibited bright green fluorescence following the addition of SYBR™ Green I, whereas negative and nontarget reactions remained orange. Positive amplification was further confirmed by a consistent amplicon annealing temperature (Ta) of 82.1 °C.

The specificity of the *P. cubensis* LAMP primers was assessed using 1.0 ng of DNA extracted from two closely related oomycete pathogens, *P. humuli* and *Peronospora belbahrii*, and DNA extracted from healthy squash leaf tissue was used as a healthy control. Only *P. cubensis* isolates showed positive reaction with LAMP, while no amplification was observed among the other isolates and control samples (Figure 3). Amplification was confirmed using three detection methods: agarose gel electrophoresis, Genie^®^ III real-time amplification, and visual detection with SYBR™ Green I. All three detection strategies showed positive amplification only for DNA from *P. cubensis*, but not from *P. humuli* and *Peronospora belbahrii* or the control samples (Figure 3). For further confirmation, an additional specificity test with five other known oomycete pathogens, *Phytophthora sojae*, *Phytopythium vexans*, *Pythium irregulare*, *Pythium dissotocum* and *Phytophthora sansomeana*, and one ascomycete pathogen, *Blumeria graminis*, produced the same result (Figure 4). Following amplification using 1 ng for each of those DNA samples, only the *P. cubensis* DNA was amplified with a Genie^®^ III real-time curve, whereas the other samples showed no amplification (Figure 4C). The Genie^®^ III real-time fluorescence outputs were entirely consistent with both our agarose gel electrophoresis and SYBR™ Green I staining results (Figure 4A,D). Samples containing *P. cubensis* DNA produced a distinct, bright green fluorescence when treated with the SYBR™ Green I dye under UV exposure, contrasting sharply with the original orange tint seen in negative and nontarget samples (Figure 4D). Furthermore, these positive samples displayed the signature ladder-like banding upon gel electrophoresis, which was completely absent in the negative controls and nontarget oomycete lanes (Figure 4A).

### 3.4. Comparative Sensitivity Analysis of LAMP Compared to PCR

**Figure 5:**
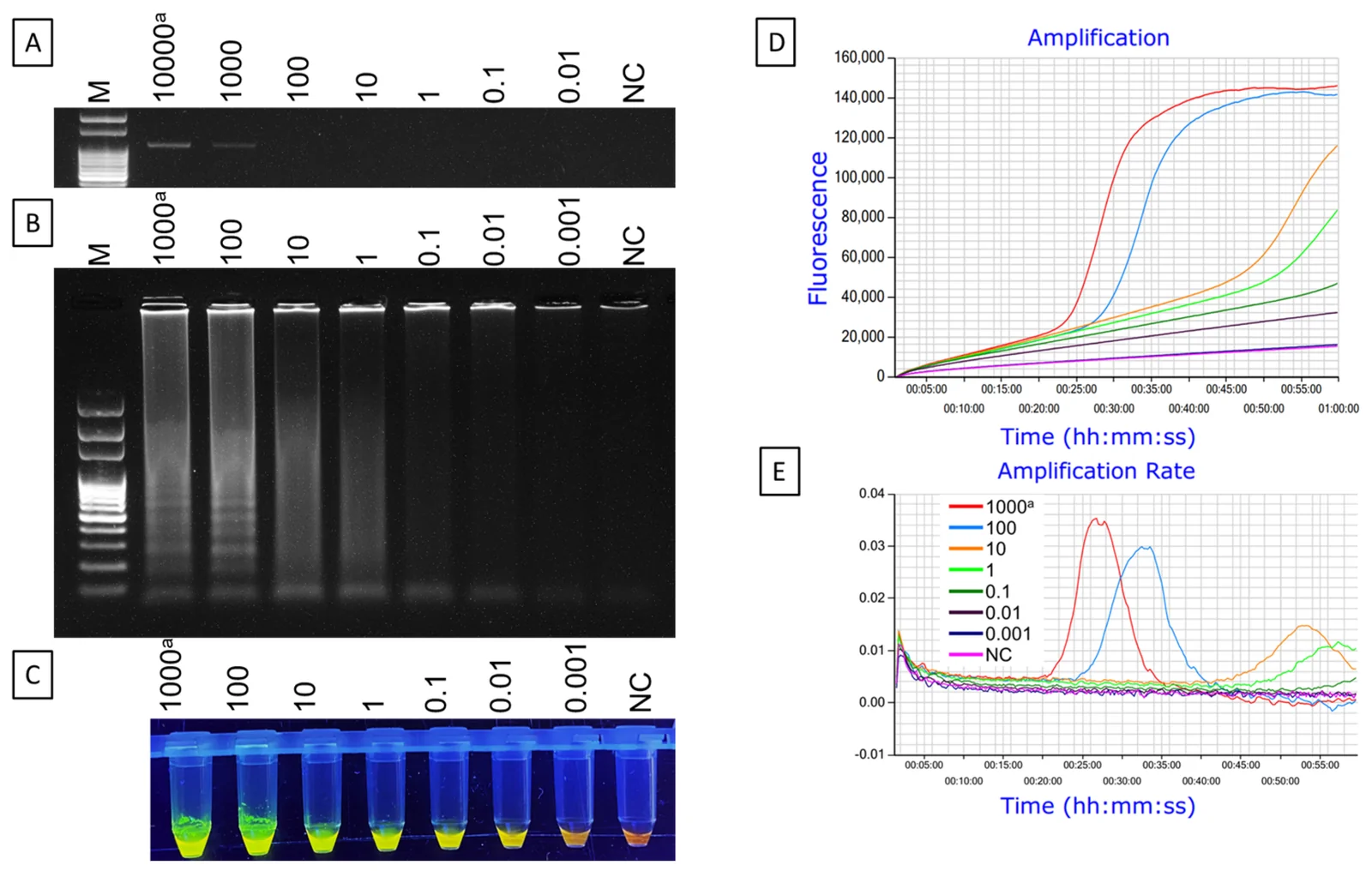
Sensitivity of the LAMP assay for the detection of *P. cubensis* using DNA from *P. cubensis* isolate C1. PCR amplification products obtained from serially diluted DNA are shown in (**A**,**B**) agarose gel electrophoresis, (**C**) visual inspection with SYBR™ Green 1 DNA gel staining agent, and (**D**,**E**) real-time amplification using Genie^®^ III. Panels (**D**,**E**) show the amplification curve and amplification rate obtained using the Genie^®^ III system. Here, a: serially diluted DNA from 10,000 pg/µL to 0.001 pg/µL; M: 100 bp DNA ladder; NC: nuclease-free H_2_O used as a negative control. The fluorescent green-colored products could be visualized after adding SYBR™ Green 1 DNA gel staining agent to the end product under UV light while negative reactions remained orange in color. Positive amplification reactions were confirmed by a consistent amplicon annealing temperature (Ta) of 82.1 °C.

A comparative sensitivity analysis of the LAMP assay and PCR was performed using a series of 10-fold dilutions of total DNA (10 ng/µL to 1.0 fg/µL) extracted from *P. cubensis* isolate C1. For PCR detection, primer set c2555.3e7_F/c2555.3e7_R (Table 2) was selected because it was more sensitive than the other primer set tested. Using the DNA dilution series, PCR successfully amplified the target down to a template concentration of 1 ng/µL. In contrast, the LAMP assay consistently detected *P. cubensis* DNA at concentrations as low as 100 pg/µL, producing distinct sigmoidal amplification curves with an optimal peak time of 32 min 30 s (Figure 5A,D,E; Table S2). Although a delayed and incomplete amplification curve was observed at 10 pg/µL (Table S2), this amplification was not considered reliable for positive detection because it was weak and inconsistent. Similarly, SYBR™ Green I staining revealed faint fluorescence at DNA concentrations as low as 1 pg/µL (Figure 5C). However, these low-concentration signals were not consistently supported by the real-time amplification curves and agarose gel electrophoresis. Therefore, the practical detection limit of the LAMP assay was defined as 100 pg/µL, based on consistent detection by the Genie^®^ III amplification curves together with confirmation by gel electrophoresis. Accordingly, the LAMP assay was at least 10-fold more sensitive than the PCR assay for detecting *P. cubensis* DNA. The results were reproducible across three independent experiments, with no discrepancies observed among the replicates.

### 3.5. Detection of P. cubensis-Infected Field-Grown and Spore-Trap Samples 

**Figure 6:**
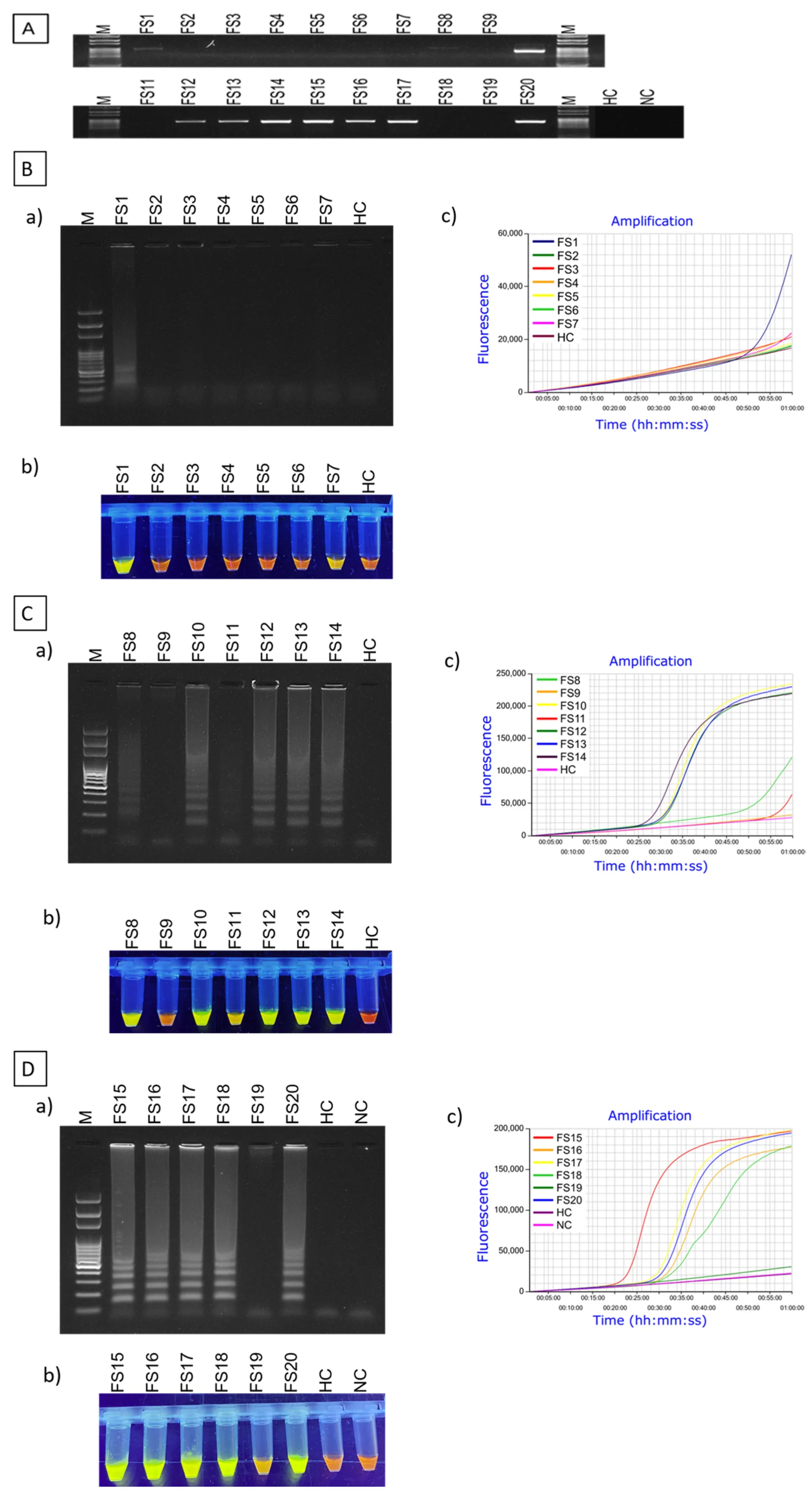
Detection of *Pseudoperonospora cubensis* in field-collected samples using conventional PCR and LAMP assays. (**A**) Conventional PCR detection of *P. cubensis* in 20 field-collected samples (FS1–FS20). M, 100-bp DNA ladder; HC, healthy control; NC, negative control. LAMP detection of *P. cubensis* in field-collected samples was evaluated by (**a**) agarose gel electrophoresis, (**b**) visual inspection using SYBR^TM^ Green I DNA gel stain, and (**c**) real-time amplification using the Genie^®^ III platform. (**B**) LAMP detection of *P. cubensis* in seven field-collected samples (FS1–FS7). (**C**) LAMP detection of *P. cubensis* in seven field-collected samples (FS8–FS14). (**D**) LAMP detection of *P. cubensis* in six field-collected samples (FS15–FS20). For the LAMP assays, HC represents healthy squash leaf samples, and NC represents dH_2_O as the negative control. Positive amplification reactions were confirmed by a consistent amplicon annealing temperature (*Ta*) of 82.1 °C. No amplification was detected in negative samples; therefore, no annealing temperature (*Ta*) was generated.

To compare the diagnostic performance of the LAMP assay and conventional PCR for detecting *P. cubensis*, 20 suspected winter squash leaf samples were collected from Tift and Lowndes counties, Georgia, USA. Field samples were classified as positive, suspected positive, or negative according to the criteria described in Section 2.9. Of the 20 samples tested, 11 were found positive by the LAMP assay, whereas 9 were found positive by conventional PCR. In addition, one sample was classified as suspected positive by the LAMP assay, while one sample was classified as suspected positive by conventional PCR (Figure 6, Table 3). Furthermore, 20 Burkard spore-trap samples obtained from the Vegetable Laboratory, Department of Plant, Soil and Microbial Sciences, Michigan State University, were also analyzed using both assays.

Overall, the results suggest that the LAMP assay may provide greater sensitivity than conventional PCR for detecting *P. cubensis* under the conditions evaluated in this study. However, the field validation was based on a limited number of positive samples, and the discordant results between LAMP and PCR were not further evaluated by sequencing or an independent molecular method such as qPCR. Therefore, additional studies involving larger sample sizes and independent confirmatory methods are needed to further validate the diagnostic performance of the LAMP assay.

## 4. Discussion

CDM is considered one of the most destructive foliar diseases affecting cucurbit hosts worldwide and is a major concern in the southeastern United States [5,33]. Early detection of this notorious pathogen is key for predicting the risk of disease incidence and to optimize the timing of fungicide applications for disease management. However, detection of *P. cubensis* is critical, as *P. humuli*, the hop downy mildew pathogen, is a very closely related species [1]. To date, several molecular-based detection markers have been utilized to identify the pathogen and differentiate it from its sister species *P. humuli*. Recently, real-time PCR assays have been utilized extensively to diagnose this pathogen, because these methods are considered a relatively rapid gold-standard approach. A set of qPCR primers was developed targeting single nucleotide polymorphisms (SNPs) within the β-tubulin genes of *P. cubensis* and *P. humuli* [23]. Based on real-time PCR amplification and high-resolution melting (HRM) analysis, *P. cubensis* was specifically identified from infected cucumber leaves [23]. Another qPCR-based improved *P. cubensis* sporangial detection system was utilized to distinguish from *P. humuli* sporangial samples captured on a Burkard spore trap [27]. In addition with these singleplex qPCR assays, identification of cucurbit downy mildew pathogen *P. cubensis* through multiplex qPCR assays based on species- and clade-specific nuclear genomic markers has recently been reported [26]. However, all these assays require advanced laboratory settings, which makes these techniques impractical for pathogen detection under field conditions. Therefore, we developed a unique isothermal technique for rapid cucurbit downy mildew detection, thereby facilitating direct, in-field diagnostic capabilities. The LAMP assay described here is an accurate, simple, sensitive, and portable diagnosis method, which could be leveraged in laboratory and field conditions for timely detection of *P. cubensis*. 

In recent years, next-generation sequencing (NGS) approaches have provided powerful approaches for identifying species-specific diagnostic markers [22,34]. Withers et al. [22] leveraged NGS data along with comparative genomics analyses to identify novel and unique genomic regions to develop diagnostic markers for the cucurbit downy mildew pathogen *P. cubensis*. Seven diagnostic markers were developed utilizing RNA-seq and DNA-seq data to identify diagnostic candidates with differential expression and to verify the presence or absence of an exon or a gene at the DNA level between two sister species, *P. cubensis* and *P. humuli* [21]. These markers were validated using PCR assays which require a well-equipped laboratory setting. Conventional PCR and Sanger sequencing-based methods are reliable; however, these techniques require substantial processing time. In this study, exploiting two single-copy marker genes, c2555.2e1 and c2555.3e7, from Withers et al. [22], we developed a LAMP assay to overcome the limitations of the above-mentioned assays. In this study, we developed a species-specific LAMP assay targeting the c2555.2e1 marker for rapid detection of *P. cubensis*, with specificity evaluated against the closely related species *P. humuli*. Among the three LAMP primer sets designed in this study (Supplementary File S1), the most suitable set of six LAMP primers successfully amplified the target *P. cubensis* DNA. It is important for a diagnostic assay to be highly specific and sensitive enough to detect a target species when it is present in low amounts [35,36,37]. The newly developed LAMP primer set was specific for the amplification of the *P. cubensis* marker gene sequence without nontarget amplification when tested with two other closely related downy mildew pathogens and six other oomycete and ascomycete fungal species (Figure 3 and Figure 4; Figure S1). Under the optimized reaction conditions at 71 °C, the LAMP assay consistently detected *P. cubensis* DNA at concentrations as low as 100 pg/µL, whereas conventional PCR reliably detected DNA down to 1 ng/µL, demonstrating that the LAMP assay was at least 10-fold more sensitive (Figure 5, Table S3). Although the assay was run for 60 min to provide adequate time to amplify, infected samples could be detected in under 25 min, while PCR amplification requires more than 2 h for the detection of the pathogen. Validation of diagnostic assays using field samples is crucial for confirming successful target amplification and the absence of cross-reactivity in plant and environmental samples. In this study, evaluation of field-infected cucurbit samples and spore-trap samples demonstrated successful amplification of *P. cubensis*, whereas no amplification was observed in healthy or negative controls. Furthermore, the LAMP assay detected a greater number of positive field samples than conventional PCR, supporting its higher analytical sensitivity under the conditions evaluated in this study (Figure 6, Table 3).

In summary, our findings support the utility of LAMP as a rapid and practical tool for plant pathogen diagnostics, highlighting its sensitivity, specificity, and ease of use in both laboratory and field settings [30,31,32]. Assay results can be interpreted using multiple detection approaches, including real-time fluorescence monitoring with the Genie^®^ III system, direct visual assessment using SYBR™ Green I dye, and conventional agarose gel electrophoresis (Figure 2, Figure 3 and Figure 4). Given the increasing severity and evolving epidemiology of *P. cubensis* outbreaks, implementation of this simple and rapid diagnostic approach could enhance early detection and support more timely and effective disease management strategies. 

## Figures and Tables

**Table 1:** Fungal isolates used for genomic DNA extraction in this study.

Fungal Species	Isolate Name	Host	Location of Collection	Source
*P. cubensis*	C1	*Cucurbita moschata* (butternut squash)	South Carolina	Quesada
*P. cubensis*	C2	*Cucumis sativus* (cucumber)	Kinston, North Carolina	Quesada
*P. humuli*	H1	*Humulus lupulus* (Hop)	Mills River, North Carolina	Quesada
*P. humuli*	H2	Hop	Mills River, North Carolina	Quesada
*Peronospora belbahrii*	B1	*Ocimum basilicum* (basil, sweet)	Clayton, North Carolina	Quesada
*Peronospora belbahrii*	B2	*Basil* (sweet)	Chapel Hill, North Carolina	Quesada
*Blumeria graminis*	SW1	*Bromus catharticus* (rescue grass)	Lowndes county, Valdosta, GA	Ali
*Phytophthora sojae*	OH.16.Eg7.lb	*Glycine max* (soybean)	Wooster, OH	Dorrance
*Phytopythium vexans*	Phyto Vex	*Citrus*	Wooster, OH	Dorrance
*Pythium irregulare*	339	*Soybean*	Wooster, OH	Dorrance
*Pythium dissotocum*	342	*Oryza sativa* (rice)	Wooster, OH	Dorrance
*Phytophthora sansomeana*	367	*Zea mays* (corn)	Wooster, OH	Dorrance

**Table 2:** Oligonucleotide sequences used for PCR and LAMP in this study.

Assay	Primers	Sequence (5’-3’)	Product Size (bp)	Purpose	Reference
PCR	c2555.2e1_F c2555.2e1_R	CGCGCAACCTTGAATACCTAC CACCCGCTCGCAGTAATGAC	831	Sequencing	[22]
	c2555.3e7_F c2555.3e7_R	GACGCAACCCAGCATAACAAG TGCGCTCGAGTATCTTCTACC	787	Sequencing, Detection	[22]
LAMP (Set 1)	F3 B3 FIP BIP LF LB	TCGAGCACATTGATGGCG CGCAGTCTCACGTACGGT TTGCACTGGATGCTCACACCCTTTTCAGCTCTTCTTTCCCGCTAG CGGTGGCAAGGCTTTGCTAGGTTTTTAATGTTCCTGGCTGCGC CCTCCTAACGAAGGAAGCCT GAATGAACAGACGAGCGAGG	NA	Detection	This study
LAMP (Set 2: Best)	F3 B3 FIP BIP LF LB	GTACCATGACAAGCGCCTC TCAACGTCATGGATGCTGAA TCGTTTGGCACCCAGATGTGCTTTTGAAAACGCGCGTGTGAAC CGCCGTAGATCTCCTCCAACGTTTTCCACAGATCCAAGTCCGC TGACCATGATTAGTCCACTGCT CATTTCCGTCGTAGCCCAC	NA	Detection	This study
LAMP (Set 3)	F3 B3 FIP BIP LF LB	AGTGCAGTCATTGGTCAACA CTCTTGACGCTGCTTGGC AGGGTCTGTCGTTGCACTCCATTTTTGTAGCCGACAAAGTTGCTCA CCGAAACTGGTCTGCCCGAATTTTTAGTCCTCGACGTCACGG ACGACCGGGGCAATGCA GAGATGGTCGAGAAGATACGTGG	NA	Detection	This study

**Table 3:** Comparison of conventional PCR and LAMP assay for the detection of *P. cubensis*-infected winter squash field samples.

Sample No.	County (GA)	Detection by PCR	Detection by LAMP	Annealing Temperature (Ta)
FS1	Tift	Positive	Positive	82.1 °C
FS2	Tift	Negative	Negative	ND
FS3	Tift	Negative	Negative	ND
FS4	Tift	Negative	Negative	ND
FS5	Tift	Negative	Negative	ND
FS6	Tift	Negative	Negative	ND
FS7	Tift	Negative	Negative	ND
FS8	Tift	Suspected positive *	Positive	82.1 °C
FS9	Tift	Negative	Negative	ND
FS10	Tift	Positive	Positive	82.1 °C
FS11	Lowndes	Negative	Suspected positive *	82.1 °C
FS12	Lowndes	Positive	Positive	82.1 °C
FS13	Lowndes	Positive	Positive	82.1 °C
FS14	Lowndes	Positive	Positive	82.1 °C
FS15	Lowndes	Positive	Positive	82.1 °C
FS16	Lowndes	Positive	Positive	82.1 °C
FS17	Lowndes	Positive	Positive	82.1 °C
FS18	Lowndes	Negative	Positive	82.1 °C
FS19	Lowndes	Negative	Negative	ND
FS20	Lowndes	Positive	Positive	82.1 °C

* Suspected positive result based on weak, delayed, or inconsistent amplification that did not fully meet the criteria for a confirmed positive result in the conventional PCR or the LAMP assay. ND, no amplification detected; therefore, no annealing temperature (Ta) was generated.

## Data Availability

The original contributions presented in this study are included in the article/Supplementary Material. Further inquiries can be directed to the corresponding author.

## Supplementary Materials

The following supporting information can be downloaded at https://www.mdpi.com/article/10.3390/jof12080626/s1: Figure S1. Screening of three LAMP primer sets for the detection of *Pseudoperonospora cubensis*. Table S1. LAMP temperature optimization using the real-time LAMP detection system Genie^®^ III. Selected temperature for LAMP amplification is highlighted in bold. Table S2. Sensitivity analysis of LAMP using the real-time LAMP detection system Genie^®^ III. The marginal nucleic acid concentration for true LAMP amplification is highlighted in bold. Table S3. BLASTN search of candidate marker genes against the NCBI nucleotide collection (nr/nt) and closely related oomycetes and ascomycetes whole genome shotgun contigs (wgs) databases. The only matched sequence from wgs database is marked with red color.

## Abbreviations

The following abbreviations are used in this manuscript:

BIP	Backward Inner Primer
CDM	Cucurbit Downy Mildew
CesA2	Cellulose synthase 2
DNA	Deoxyribonucleic Acid
FIP	Forward Inner Primer
GA	Georgia
HDM	Hop Downy Mildew
HRM	High-Resolution Melting
ITS	Internal Transcribed Spacer
LAMP	Loop-Mediated Isothermal Amplification
LB	Loop Backward Primer
LF	Loop Forward Primer
NCBI	National Center for Biotechnology Information
NGS	Next-Generation Sequencing
PCR	Polymerase Chain Reaction
qPCR	Quantitative Polymerase Chain Reaction
RNA	Ribonucleic Acid
SNPs	Single Nucleotide Polymorphisms
Ta	Amplicon Annealing Temperature
Tiamp	Amplification Time
UGA-MDL	University of Georgia Plant Molecular Diagnostic Lab
US	United States
UV	Ultraviolet
