# Supplementary material for: Development of a Novel Pseudoperonospora cubensis-Specific LAMP Assay for Rapid Field Detection and Surveillance of Cucurbit Downy Mildew
Source: J Fungi (Basel). 2026 Aug 20;12(8):626. doi: 10.3390/jof12080626 (PMC13514291; doi:10.3390/jof12080626)
Supplement: Supplementary file 1 [file jof-12-00626-s001.zip › jof-4443486-supplementary.pdf]

Table S1. LAMP temperature optimization using the real-time LAMP detection system Genie® III. Selected temperature for LAMP amplification is highlighted in bold.

Tm_{amp} (°C)[a]	Ti_{amp} (Min:S)[b]	Ta (°C)[c]
66	50:45	82.3
67	44:00	82.4
68	40:15	82.4
69	32:45	82.4
70	28:00	82.5
**71**	**25:00**	**82.1**
72	25:30	82.1
73	26:45	82.5

Amplification temperature in degree Celsius (°C)

[b]Amplification time in minutes and seconds (Min:S)

[c]Annealing temperature in degree Celsius (°C)

Table S2. Sensitivity analysis of LAMP using the real-time LAMP detection system Genie® III. The marginal nucleic acid concentration for true LAMP amplification is highlighted in bold.

Name of well	C_{amp} (pg/µl)[a]	Ti_{amp} (Min:S)[b]
1	1000	26:45
**2**	**100**	**32:30**
3	10	53:15
4	1	N/A
5	0.1	N/A
6	0.01	N/A
7	0.001	N/A
Neg	0	N/A

Table S3. BLASTN search of candidate marker genes against the NCBI nucleotide collection (nr/nt) and closely related oomycetes and ascomycetes whole genome shotgun contigs (wgs) databases . The only matched sequence from wgs database is marked with red color.

Candidate marker	Name of organism	NCBI standard database				
		Nucleotide collection (nr/nt)	Blast result	Whole genome shotgun contigs (wgs)	Link to database	Blast result
c2555_2e1 and c2555_3e7	All	Whole database	No hit	N/A	N/A	N/A
	Pseudoperonospora cubensis	*P. cubensis* (taxid:143453)	No hit	*P. cubensis* (taxid:143453)	WGS_VDB://AHJF01	*P. cubensis* isolate MSU-1 psc_contig_2555, whole genome shotgun sequence, GenBank accession number: AHJF01002473.1, sequence match with c2555_2e1 marker is 99.86% and c2555_3e7 marker is 100%
	Pseudoperonospora spp.	*Pseudoperonospora* (taxid:143452)	No hit			
		unclassified *Pseudoperonospora* (taxid:1000571)				
		Pseudoperonospora sp. 23231 (taxid:1000574)				
		Pseudoperonospora sp. 24230 (taxid:1000575)				
		Pseudoperonospora sp. (taxid:2169586)				
		Pseudoperonospora cannabina (taxid:291622)				
		Pseudoperonospora urticae (taxid:162119)				
		Pseudoperonospora celtidis (taxid:291623)				
		Pseudoperonospora sp. 21226 (taxid:1000572)				
		Pseudoperonospora sp. 22238 (taxid:1000573)				
		Pseudoperonospora sp. D149 (taxid:1000576)				
		Pseudoperonospora sp. D190 (taxid:1000577)				
		Pseudoperonospora sp. D232 (taxid:1000578)				
		Pseudoperonospora sp. D265 (taxid:1000579)				
	Pseudoperonospora humuli	*P. humuli* (taxid:162120)	No hit	*P. humuli* (taxid:162120)	WGS_VDB://NQFO01	No hit
	Peronospora belbahrii	*P. belbahrii* (taxid:622444)	No hit	*P. belbahrii* (taxid:622444)	WGS_VDB://CACTHD01	No hit
					WGS_VDB://MRBU01	
	Plasmopara obducens	*P. obducens* (taxid:162140)	No hit	*P. obducens* (taxid:162140)	WGS_VDB://LGJC01	No hit
		P. obducens (taxid:162140)			WGS_VDB://LGJD01	
	Phytophthora capsici		No hit		WGS_VDB://JAFLQK01	No hit
					WGS_VDB://JAFLQJ01	
					WGS_VDB://ADVJ01	
					WGS_VDB://JAFLQL01	
		P. capsici (taxid:4784)		*P. capsici* (taxid:4784)	WGS_VDB://RQEI01	
		P. capsici LT1534 (taxid:763924)		*P. capsici* Leonian (taxid:4784)	WGS_VDB://RQEJ01	
				P. capsici LT1534 (taxid:763924)	WGS_VDB://RQEK01	
					WGS_VDB://RQEL01	
					WGS_VDB://RQEM01	
					WGS_VDB://RQEN01	
	Phytophthora infestans				WGS_VDB://AATU01	No hit
		P. infestans (taxid:4787)		*P. infestans* (Mont.) de Bary 1876 (taxid:4787)	WGS_VDB://JAACNO01	
		P. infestans strain T30-4 (taxid:403677)		*P. infestans* (taxid:4787)	WGS_VDB://LYVM01	
		P. infestans T30-4 (taxid:403677)		*P. infestans* (taxid:4787)	WGS_VDB://SEHI01	
					WGS_VDB://WSZM01	
	Phytophthora sojae	*P. sojae* (taxid:67593)	No hit	*P. sojae* (taxid:67593)	WGS_VDB://AAQY02	No hit
		P. sojae strain P6497 (taxid:1094619)			WGS_VDB://WWEI01	
	Phytopythium vexans		No hit		WGS_VDB://AKYC02	No hit
		P. vexans (taxid:907947)		*P. vexans* (de Bary) Abad, De Cock, Bala, Robideau, Lodhi & Lvesque 2015 (taxid:907947)	WGS_VDB://JAADXV01	
				P. vexans (taxid:907947)	WGS_VDB://JAKLCF01	
					WGS_VDB://QLOC01	
	Pythium irregulare		No hit	*P. irregulare* (taxid:36331)	WGS_VDB://AKXZ02	No hit
		P. irregulare (taxid:36331)		*P. irregulare* DAOM BR486 (taxid:1223557)	WGS_VDB://JAADWQ01	
				P. irregulare Buisman, 1927 (taxid:36331)	WGS_VDB://NCVO01	
	Pythium dissotocum	*P. dissotocum* (taxid:82936)	No hit	*P. dissotocum* (taxid:82936)	WGS_VDB://JAADWY01	No hit
	Phytophthora sansomeana	*P. sansomeana* (taxid:555429)	No hit	N/A	N/A	N/A
		Phytophthora sp. 'kelmania' x P. sansomeana (taxid:2678298)				
	Blumeria graminis		No hit	*B. graminis* (taxid:34373)	WGS_VDB://JAJOCF01	No hit
				B. graminis f. sp. hordei (taxid:62688)	WGS_VDB://AOIY01	
		B. graminis (taxid:34373)		*B. graminis* f. sp. hordei DH14 (taxid:546991)	WGS_VDB://CAUH01	
		B. graminis f. sp. tritici (taxid:62690)		*B. graminis* f. sp. hordei strain DH14 (taxid:546991)	WGS_VDB://OENG01	
		B. graminis f. sp. triticale (taxid:1689686)		*B. graminis* subsp. f. sp. hordei (taxid:62688)	WGS_VDB://CAAAHF01	
		B. graminis f. sp. tritici 96224 (taxid:1268274)		*B. graminis* f. sp. hordei (taxid:2867405)	WGS_VDB://UNSH01	
		B. graminis f. sp. hordei strain DH14 (taxid:546991)		*B. graminis* f. sp. hordei A6 (taxid:1283758)	WGS_VDB://AOLT01	
		B. graminis f. sp. secalis (taxid:62689)		*B. graminis* f. sp. triticale (taxid:1689686)	WGS_VDB://CAJHIT01	
				B. graminis f. sp. tritici (taxid:62690)	WGS_VDB://ASJK01	
				B. graminis f. sp. tritici 96224 (taxid:1268274)	WGS_VDB://ANZE01	
				B. graminis f. sp. tritici Bgt#70 (taxid:1307830)	WGS_VDB://ASJN01	
				B. graminis f. sp. tritici JIW2 (taxid:1306134)	WGS_VDB://ASJL01	

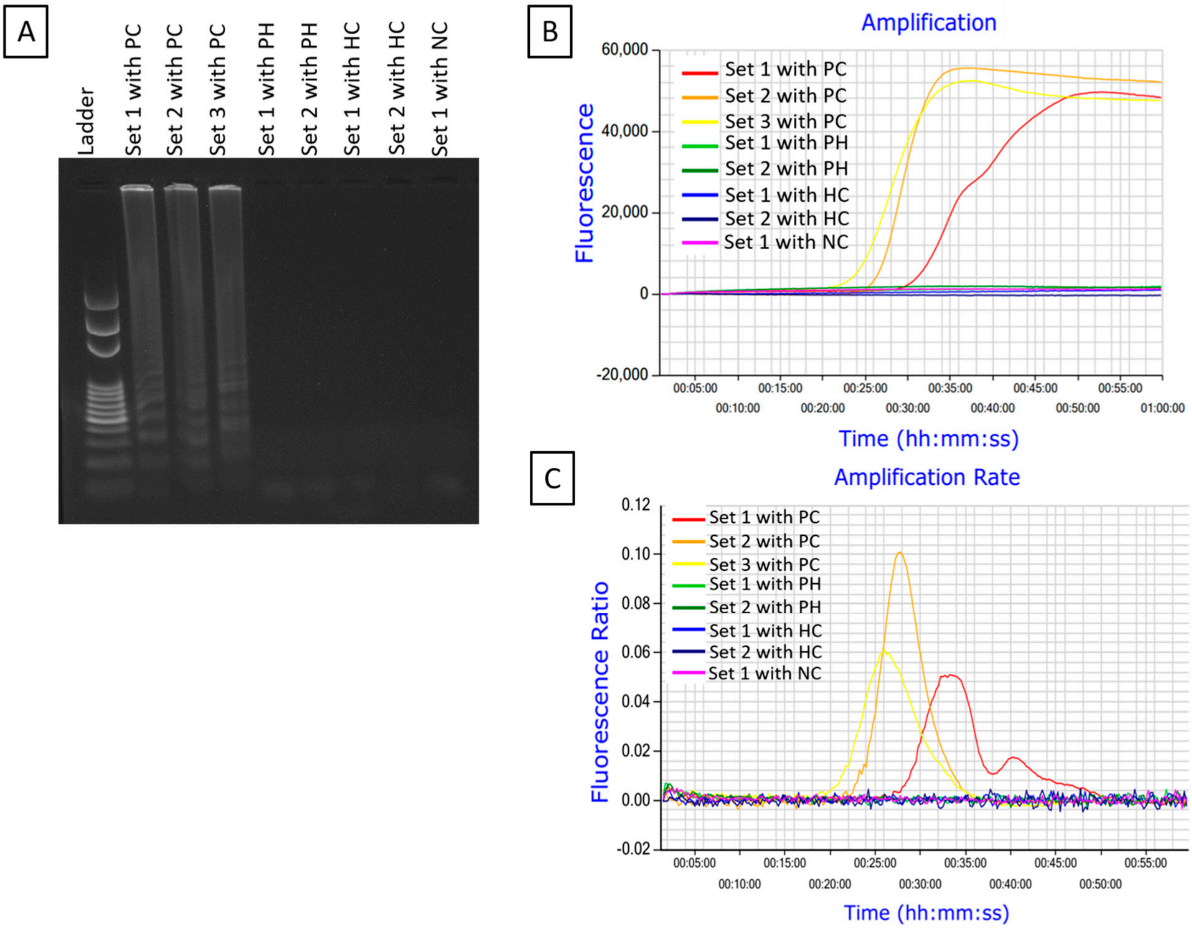

Figure S1: Screening of three LAMP primer sets for the detection of *Pseudoperonospora cubensis*. Amplification products were analyzed by (A) 1.0% agarose gel electrophoresis, (B) real-time amplification using the Genie® III system, and (C) amplification rate curves generated by the Genie® III system. LAMP Primer Sets 1, 2, and 3 were evaluated using genomic DNA from *P. cubensis* (PC). Primer Sets 1 and 2 were further evaluated using genomic DNA from *P. humuli* (PH), healthy squash leaf tissue (HC), and the no-template control (NC; nuclease-free H_2O). M, 100-bp DNA ladder.
